# Aetiological Evaluation of Oligodontia in a Three-Generation Family

**DOI:** 10.3290/j.ohpd.a44033

**Published:** 2020-07-04

**Authors:** Sezen Güntekin Ergün, Burcu Baloş Tuncer, Mehmet Ali Ergün, Kolbaşı Guyem, Metin Orhan, Ferda E Perçin

**Affiliations:** a Researcher, Department of Medical Genetics, Gazi University Faculty of Medicine, Ankara, Turkey; Department of Medical Biology, Hacettepe University Faculty of Medicine, Ankara, Turkey. Performed all experiments, assisted with obtained consents, biomaterials, confirmed co-segregation in the family, assisted with manuscript editing.; b Professor, Department of Orthodontics, Gazi University Faculty of Dentistry, Ankara, Turkey. Diagnosed and assisted the treatment of the patients, contributed to writing the manuscript.; c Professor, Department of Medical Genetics, Gazi University Faculty of Medicine, Ankara, Turkey. Analysed exome data, identified the causative mutation, assisted writing the manuscript.; d Assistant, Department of Psychiatry, Uludag University Faculty of Medicine, Bursa, Turkey. Department of Medical Genetics, Gazi University Faculty of Medicine, Ankara, Turkey; Obtained consents and/or biomaterials, and assisted with analysed patient data.; e Professor, Department of Orthodontics, Ankara Yıldırım Beyazıt University, Faculty of Dentistry, Ankara, Turkey. Diagnosed the patients.; f Professor, Gazi University Faculty of Medicine, Department of Medical Genetics, Ankara, Turkey. Designed and coordinated the project, analysed patient clinical data, assisted with analysis of molecular data, contributed with writing the manuscript.

**Keywords:** oligodontia, dental agenesis, whole-exome sequencing, WNT10A

## Abstract

**Purpose:**

The aim of this study was to assess the genetic evaluation of a three-generation consanguineous family with isolated oligodontia.

**Materials and Methods:**

A 16-year-old male patient who had been referred for orthodontic treatment due to the presence of oligodontia, and his family members who presented several missing teeth had been enrolled in the study. Clinical and radiological assessments and genetic analysis including whole-exome sequencing were performed.

**Results:**

Genetic evaluations revealed both homozygous and heterozygous mutations (c.T682A:p.F228I) in the *WNT10A* gene of six affected members of the family. Higher frequency of agenesis of mandibular second molar was found in homozygous relative to heterozygous *WNT10A* mutations.

**Conclusion:**

The present findings have provided evidence for a known variant in the *WNT10A* gene in a three-generation consanguineous family with isolated oligodontia, while the results confirmed that cases with homozygous mutation revealed clinical hetero­geneity.

Tooth agenesis (TA) is one of the most common developmental dental anomalies, which might have adverse effects on oral functions, masticatory functions, and aesthetics. These alone may cause psychological problems as well. It is an inherited feature and can be seen as isolated, or as part of a specific syndrome, such as ectodermal dysplasias. TA is defined as congenital absence of one or more primary or secondary teeth, excluding third molars, and can be classified as hypodontia (1–5 teeth missing), oligodontia (≥ 6 teeth missing) or anodontia (complete absence of teeth). The role of several disturbances and gene mutations during different stages of tooth development have been emphasised as contributors to TA.^2,6,10,18^

Since the mutations in the *MSX1* gene have been shown to cause the non-syndromic teeth agenesis, investigations showed that^14^ additionally causative genes including *PAX9*, *WNT10A*, *MSX1*, *EDA*, *LRP6*, *WNT10B*, *AXIN2*, *BMP4*, *DKK1*, *EDAR*, *EDARADD*, *GREM2*, *KREMEN1*, *LTBP3* and *SMOC2* playing role in the three signalling pathways are also responsible. The data indicate that mutations in seven of these genes (*PAX9*, *WNT10A*, *MSX1*, *WNT10B*, *LRP6*, *AXIN2*, *EDA*) are responsible for 91.9% of these cases.^2,5,6,8,10,12,15,18,19^ Authors suggested that mutated genes encoding the components in the canonical Wnt/β-catenin pathway and Wnt-associated genes have higher genetic risk for isolated TA compared to genes which play roles in other pathways.^2,5,8,10,15,18,19^ *WNT10A* variants are associated with both selective TA 4 (STHAG4), odontoonychoectodermal dysplasia (OODD) and Schopf–Schulz–Passarge syndrome (SSPS).^4^

This study presents clinical, radiological and genetic evaluations of a familial non-syndromic oligodontia caused by variation on *WNT10A* gene.

## MATERIALS AND METHODS

### Clinical Report

**Fig 1:**
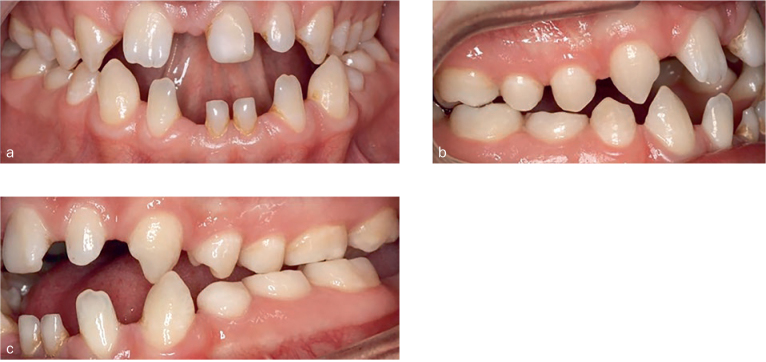
Intraoral views of the proband (OD3) demonstrating the malocclusion

**Fig 2:**
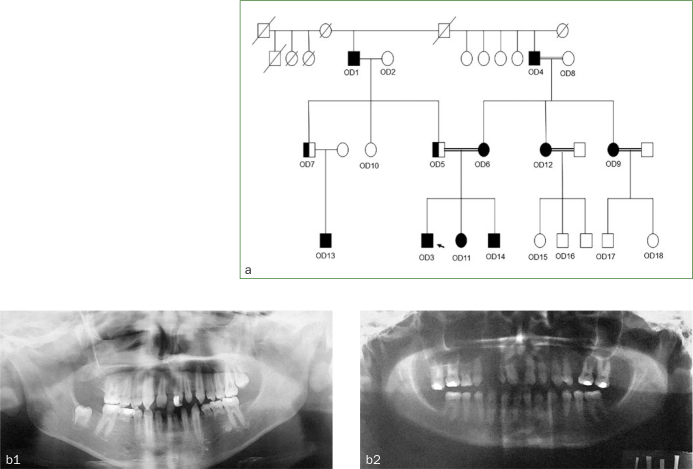
(a) Pedigree of the family. (b) Panoramic radiographs of patients positive for *WNT10A* mutations, revealing homozygous mutations in OD3 and OD6.

A male patient (16 years 8 months) was referred with a main complaint of difficulty in biting, speech problems and aesthetic concerns due to severe diastema (Fig 1). There was a history of consanguineous marriages in the family and most of the family members had numerous teeth agenesis. The family was from Ankara, Turkey. The pedigree consisted of a total of 33 people, of whom nine were affected in four generations (Fig 2a). Seven family members had already died by the time of the study. Sixteen family members were intraorally examined by a dentist. All the affected members of the family were also physically examined by a clinical geneticist. A total of 18 blood samples were obtained and DNA extracted from relevant family members for molecular evaluation, but only 8 of them were analysed. The molecular genetic analysis of the family was performed at the Department of Medical Genetics, Faculty of Medicine, Gazi University in between 2012 and 2016. All the participants or their guardians signed informed consent forms and the study protocol was approved by the local Ethic Committee of Gazi University with a reference number of 2012/021.

### Molecular Analysis

Whole-exome sequencing (WES) analysis was only performed to the proband (OD3). The result of WES analysis was confirmed by Sanger sequencing (OD3). Mutation screening was also performed in five other affected (OD5, OD6, OD7, OD11, OD14) and two healthy (OD8, OD15) family members.

### DNA Isolation

A 5 ml peripheral blood sample was collected with consent from each patient in an EDTA tube. The isolation of the DNA was performed with the NucleoSpin Blood kit (Macherey-Nagel, Düren, Germany) according to the manufacturers’ protocol. The concentration and quality of eluted DNA sample was analysed by a spectrophotometer (NanoDrop ND 1000, USA).

### Whole-Exome Sequencing (WES)

Regarding the WES analysis the enrichment was performed by Nextera Rapid Capture Expanded Exome Kit with the Illumina HiSeq platform with a coverage of ×70 (TUBITAK-MAM). The analysis was performed by the Arpeggi Engine. This pipeline has been used for alignment, variant calling, and variant annotation. The raw data had been provided in VCF format.

### Analysis of Sequence Variations

After annotating the VCF data with the web interface to the ANNOVAR software (wANNO-VAR) www.wannovar.usc.edu/, the annotated data has been transferred to MS Excel file. This data had been analysed by an in-house workflow named SELIM. SELIM had been reported to be constructed in order to filter and prioritise the candidate variants across individual patients and healthy controls that have been subjected to WES in eight steps. This method was reported to be based on to filter the variants with respect to an algorithm without using in silico tools.^7^ In our case the raw data was composed of 315,782 variants and with SELIM the data was decreased to 1469 variants.

### Sanger Sequencing

We designed primers with an online tool, Primer3 (v. 0.4.0), which is used for designing polymerase chain reaction (PCR) primers.^3^ The sequencing results were then aligned with the reference gene sequences available in the NCBI database. Sequencing reactions were conducted using Genetic Analyzer 310 (ABI/Life Technologies).

## RESULTS

Intraoral clinical examination of the proband (OD3) showed Angle Class I malocclusion at the right and Angle Class II malocclusion at the left side. There were polidiastema in both arches, unilateral cross-bite and retained five primary teeth (71, 74, 75, 81, 85). No presence of macrodontia or enamel hypoplasia was found. Overjet was 0 mm, and there was a 3 mm open bite (Fig 1). The labiolingual bone thickness was decreased in mandibular anterior region. Radiographic findings revealed no presence of endodontic problems, but agenesis of 17, 12, 31, 34, 35, 37, 41 and 45 was found (Fig 2b1). Physical examination revealed no abnormality of hair or nails, and sweating was normal. Due to the finding that most of the family members had numerous missing teeth, clinical and radiological evaluations were repeated with other family members (Fig 2b2). Number of missing teeth for the whole family has been recorded (Table 1).

**Table 1:** Clinical and molecular evaluations in relation to missing teeth

ID	Congenitally absent teeth		WNT10A mutations
	Maxilla	Mandible	
OD-1	NA*	NA*	NA
OD-2	None	None	NA
OD-3	12, 17	31, 34, 35, 37, 41, 45	Homozygous
OD-4	None	31, 32, 41, 42	NA
OD-5	NA*	NA*	Heterozygous
OD-6	12, 14, 15, 25	37, 47	Homozygous
OD-7	12, 22	None	Heterozygous
OD-8	None	None	NA
OD-9	None	31, 32, 41, 42	NA
OD-10	None	None	NA
OD-11	12, 22	31, 41	Homozygous
OD-12	NA*	NA*	NA
OD-13	12, 22	None	NA
OD-14	NA**	NA**	Homozygous
OD-15	None	None	Wild type
OD-16	None	None	Wild type

ID, DNA numbers of the patients; NA, not applied; * Presence of total prosthesis, ** Too young for evaluation.

Evaluation of the responsible genes for oligodontia was performed, and a homozygous missense mutation (c.T682A:p.F228I) in exon3 of *WNT10A* gene of the proband (OD3) was found. Sanger sequencing results also confirmed these results in proband (OD3). This mutation was analysed with MutationTaster, an in silico analysis program for mutation prediction, and was found to be disease-causing.^13^ The same homozygous mutation was found in three of the five other patients in the family (OD6, OD11, OD14), whereas the mutation was heterozygous in the other two members (OD5, OD7). The results from the healthy individuals were normal (OD8, OD15) (Table 1).

## DISCUSSION

Oligodontia, a severe form of TA, is a genetically and phenotypically heterogeneous condition. Diagnosis first involves a medical and dental history, followed by a detailed intraoral and radiographical examination of the affected family members. Counselling with a geneticist is essential to identify the association with syndromes or other possible anomalies, and the genetic basis of the anomaly. Syndromic and non-syndromic forms of oligodontia can be differentiated by conducting physical examination of hair, nails, sweat glands, eyes, and presence of any congenital disorders. Besides, drawing pedigree might be beneficial in determining the pattern of inheritance.

To date, several mutations in 15 genes have been detected in familial TA while most of the cases were related with mutations in the *WNT10A* gene.^2,5,8,15,18,19^ TA with or without ectodermal dysplasia is caused by homozygous, heterozygous, or compound heterozygous mutation in the *WNT10A* gene in more than 50% of patients with oligodontia.^8,9,15-19^ Typically, homozygous mutations in *WNT10A* cause various ectodermal dysplasia syndromes often corresponding to odontoonychodermal dysplasia (OODD; MIM #257980) and SSPS (MIM #224750), both including classic ectodermal developmental anomalies such as hypo/oligodontia, nail dysplasia, lacrimal duct hypo/aplasia, hypohidrosis, and hypotrichosis with additional cutaneous features. SPSS is distinguished by the presence of multiple eyelid cysts, while OODD is apparently characterised by hypoplasia of lingual papillae.^4,8^ In the present study, we found homozygote or heterozygote missense mutation c.682T>A (p.F228I) in the *WNT10A* gene of six affected members of the family with oligodontia without any ectodermal features. Correlations with *WNT10A* molecular status (heterozygous carrier, compound heterozygous, homozygous) and patient’s phenotypes has been the focus of many studies. While in patients bearing biallelic *WNT10A* mutations including compound heterozygous and homozygous showed a marked phenotypic variability, heterozygous carriers have milder dental phenotypes.^1,2,15-17^ Anomalies in tooth morphology were frequently observed in patients with heterozygous mutations.^15^ Also, heterozygous genotypes for some mutations of *WNT10A* have also been found in ~2.3% of unaffected controls.^6^ Arzoo et al showed that homozygous *WNT10A* mutations were associated with a higher frequency of molar and mandibular central incisor agenesis relative to heterozygous *WNT10A* mutations.^2^ In relation to current findings, mandibular central incisor agenesis was detected in two siblings with homozygous mutation (OD3, OD11), while their mother carrying the homozygous mutation (OD6) showed no agenesis of mandibular central incisors. Again, the number of missing teeth and pattern of TA declared differences between the proband (OD3), his sister (OD11) and the mother (OD6). Thus, clinical heterogeneity has been observed in our cases, even in individuals carrying homozygous mutation. In our study, both homozygote and heterozygote members commonly lacked maxillary lateral incisors and showed reduced teeth size with conical form for anterior teeth. Absence of second molars and second premolars, either in the maxillary or mandibular arch, and missing mandibular central incisors were also common in homozygotes. Thus, our clinical results confirmed the presence of multiple numbers of missing teeth in the case of homozygous mutations.

Results of a meta-analysis revealed that the mandibular second premolar was the most affected tooth, followed by the maxillary lateral incisor and maxillary second premolar.^11^ However, maxillary lateral incisors were commonly affected in the present family members. Agenesis of maxillary central incisors were reported to be extremely rare.^11^ Similarly, none of the affected family members in this study lacked maxillary central incisors.

## CONCLUSION

Taken together, the molecular mechanisms for the expression of different genes and proteins in tooth formation is very complex. In accordance with the previous reports, the present findings support the association between *WNT10A* mutation and non-syndromic oligodontia. Oligodontia is an aesthetically and functionally disturbing problem for the patients and their parents, since this problem requires interdisciplinary treatment protocols, which may be compelling for the families. Although it requires a multidisciplinary approach and multiple tests, evaluation of family members and drawing pedigree will be useful in understanding the genetic transition of such problems. Therefore, such evaluations may not only provide beneficial knowledge for the clinicians, but will also be helpful for enlightening the concerns of the families.

## References

[ref1] Abid MF, Simpson MA, Barbosa IA, Seppala M, Irving M, Sharpe PT (2018). WNT10A mutation results in severe tooth agenesis in a family of three sisters. Orthod Craniofac Res.

[ref2] Arzoo PA, Klar J, Bergendal B, Norderyd J, Dahl N (2013). WNT10A mutations account for 1/4 of population-based isolated oligodontia and show phenotypic correlations. Am J Med Genet Part A.

[ref3] Choi M, Scholl UI, Ji W, Liu T, Tikhonova IR, Zumbo P (2009). Genetic diagnosis by whole exome capture and massively parallel DNA sequencing.

[ref4] (2018). www.omim.org/.

[ref5] Dhamo B, Fennis W, Créton M, Vucic S, Cune M, van Amstel HKP (2016). The association between WNT10A variants and dental development in patients with isolated oligodontia. Eur J Human Genet.

[ref6] Dinckan N, Du R, Petty LE, Coban-Akdemir Z, Jhangiani SN, Paine I (2018). Whole-exome sequencing identifies novel variants for tooth agenesis. J Dent Res.

[ref7] Ergun MA, Unal A, Guntekin Ergun S, Percin EF (2017). A new method for analysis of whole exome sequencing data (SELIM) depending on variant prioritization. Informatics in Medicine Unlocked.

[ref8] Jose van den Boogaard M, Creton M, Bronkhorst Y, van der Hout A, Hennekam E, Lindhout D (2012). Mutations in WNT10A are present in more than half of isolated hypodontia cases. J Med Genet.

[ref9] Kantaputra P, Sripathomsawat W (2011). WNT10A and isolated hypodontia. Am J Med Genet.

[ref10] Massink MPG, Creton MA, Spanevello F, Fennis WMM, Cune MS, Sanne MC (2015). Loss-of-function mutations in the WNT co-receptor LRP6 cause autosomal-dominant oligodontia. Am J Hum Genet.

[ref11] Polder BJ, Van’t Hof MA, Van der Linden FP, Kuijpers-Jagtman AM (2004). A meta-analysis of the prevalence of dental agenesis of permanent teeth. Community Dent Oral Epidemiol.

[ref12] Ruiz-Heiland G, Jabir S, Wende W, Blecher S, Bock N, Ruf S (2016). Novel missense mutation in the EDA gene in a family affected by oligodontia. J Orofac Orthop.

[ref13] Schwarz JM, Cooper DN, Schuelke M, Seelow D (2014). MutationTaster2: mutation prediction for the deep-sequencing age. Nat Methods.

[ref14] Suomalainen M, Thesleff I (2010). Patterns of Wnt pathway activity in the mouse incisor indicate absence of Wnt/beta-catenin signaling in the epithelial stem cells.

[ref15] Tardieu C, Jung S, Niederreither K, Prasad M, Hadj-Rabia S, Philip N (2017). Dental and extra-oral clinical features in 41 patients with WNT10A gene mutations: a multicentric genotype–phenotype study. Clin Genet.

[ref16] van den Boogard MJ, Creton M, Bronkhorst Y, van der Hout A, Hennekam E, Lindhout D (2012). Mutations in WNT10A are present in more than half of isolated hypodontia cases. J Med Genet.

[ref17] Vink CP, Ockeloen CW, ten Kate S, Koolen DA, van Amstel JKP, Jagtman AMK (2014). Variability in dentofacial phenotypes in four families with WNT10A mutations. Eur J Human Genet.

[ref18] Yu M, Wong SW, Han D, Cai T (2019). Genetic analysis: Wnt and other pathways in non-syndromic tooth agenesis. Oral Dis.

[ref19] Yu P, Yang W, Han D, Wang X, Guo S, Li J (2016). Mutations in WNT10B are identified in individuals with oligodontia. Am J Hum Genet.

